# Immunogenicity and Safety of a Third COVID-19 BNT162b2 mRNA Vaccine Dose in 5- to 11-Year Olds

**DOI:** 10.1093/jpids/piad015

**Published:** 2023-03-16

**Authors:** Eric A F Simões, Nicola P Klein, Charu Sabharwal, Alejandra Gurtman, Nicholas Kitchin, Benita Ukkonen, Piotr Korbal, Jing Zou, Xuping Xie, Uzma N Sarwar, Xia Xu, Stephen Lockhart, Luke Cunliffe, Claire Lu, Hua Ma, Kena A Swanson, Kenneth Koury, Pei-Yong Shi, David Cooper, Ӧzlem Türeci, Kathrin U Jansen, Uğur Şahin, William C Gruber

**Affiliations:** Children’s Hospital Colorado and University of Colorado School of Medicine, Aurora, CO, USA; Kaiser Permanente Vaccine Study Center, Kaiser Permanente Northern California, Oakland, CA, USA; Vaccine Research and Development, Pfizer Inc, Pearl River, NY, USA; Vaccine Research and Development, Pfizer Inc, Pearl River, NY, USA; Vaccine Research and Development, Pfizer Ltd, Hurley, UK; Espoo Vaccine Research Clinic, Tampere University, Espoo, Finland; Ośrodek Badań Klinicznych In-Vivo, Bydgoszcz−Toruń, Poland; Department of Biochemistry and Molecular Biology, University of Texas Medical Branch, Galveston, TX, USA; Department of Biochemistry and Molecular Biology, University of Texas Medical Branch, Galveston, TX, USA; Vaccine Research and Development, Pfizer Inc, Pearl River, NY, USA; Vaccine Research and Development, Pfizer Inc, Collegeville, PA, USA; Vaccine Research and Development, Pfizer Ltd, Hurley, UK; Vaccine Research and Development, Pfizer Ltd, Hurley, UK; Vaccine Research and Development, Pfizer Inc, Pearl River, NY, USA; Vaccine Research and Development, Pfizer Inc, Collegeville, PA, USA; Vaccine Research and Development, Pfizer Inc, Pearl River, NY, USA; Vaccine Research and Development, Pfizer Inc, Pearl River, NY, USA; Department of Biochemistry and Molecular Biology, University of Texas Medical Branch, Galveston, TX, USA; Vaccine Research and Development, Pfizer Inc, Pearl River, NY, USA; BioNTech, Mainz, Germany; Vaccine Research and Development, Pfizer Inc, Pearl River, NY, USA; BioNTech, Mainz, Germany; Vaccine Research and Development, Pfizer Inc, Pearl River, NY, USA

**Keywords:** COVID-19, BNT162b2 vaccine, children, booster, immunogenicity, safety

## Abstract

In this ongoing study, substantially increased ancestral SARS-CoV-2 neutralizing responses were observed 1 month after a third 10-µg BNT162b2 dose given to 5 to 11-year olds versus neutralizing responses post-dose 2. After dose 3, increased neutralizing responses against Omicron BA.1 and BA.4/BA.5 strains were also observed. The safety/tolerability profile was acceptable. (NCT04816643)

## Teaser

A third BNT162b2 10-µg vaccine dose administered approximately 6 months after the second dose to 5- to 11-year olds increased neutralizing titers, including against Omicron BA.1 and BA.4/BA.5 strains. The safety and tolerability profile of the third BNT162b2 10-µg dose was acceptable.

## INTRODUCTION

Vaccinating school-age children with a third dose (booster) of a COVID-19 vaccine is an urgent public health need as this population remains vulnerable to COVID-19 and may transmit SARS-CoV-2, particularly of highly transmissible variants. In a phase 2/3 study conducted before the Omicron wave, a BNT162b2 vaccine two-dose primary series given 21 days apart was immunogenic and 91% effective against COVID-19 in 5- to 11-year olds [1]. However, immune evasion of currently circulating Omicron variants observed in adults support the need for a third BNT162b2 dose to enhance COVID-19 protection against Omicron variants and potentially against emerging future escape variants in children [2–4]. Relative vaccine efficacy in ≥16-year olds after receiving a third 30-µg BNT162b2 dose compared with two doses was 95%, during Delta variant predominance [5]. Therefore, we evaluated whether a third 10-µg BNT162b2 dose administered approximately 6 months after dose 2 in 5- to 11-year olds was safe and tolerable and able to enhance the magnitude and breadth of neutralizing antibody responses to the SARS-CoV-2 ancestral strain and Omicron variants.

## METHODS

In this ongoing study, existing 5- to 11-year-old participants from a phase 2/3 study (NCT04816643) who received two 10-µg BNT162b2 doses given 21 days apart received an open-label third (booster) dose of 10-µg BNT162b2 at least 6 months after dose 2. The dose level of BNT162b2 doses 2 and 3 was based on the participant’s age at the time of vaccination. Therefore, participants who were 12 years old at the time of receiving the third dose are not included in this analysis as they received the recommended age-appropriate 30-µg dose level. Eligibility criteria, ethical study conduct, and study responsibilities are summarized in the Appendix.

Safety evaluations included assessment of participant- or parent/legal guardian-reported local reactions, systemic events, and antipyretic use recorded in an electronic diary for up to 7 days after dose 3. Adverse event (AE) reports were collected from dose 3 through 1 month after dose 3. Serious AEs are being collected from dose 3 through approximately 6 months after dose 3.

In this analysis, the main immunogenicity assessments were conducted in participants without prior SARS-CoV-2 infection (see Appendix for determination of prior infection status). Assessments were also conducted in participants with and without prior SARS-CoV-2 infection. Blood samples were collected for immunogenicity assessments, which included determination of SARS-CoV-2 neutralization titers with a validated assay using the ancestral (USA-WA1/2020) strain as described previously and with a non-validated Fluorescent Focus Reduction Neutralization Test (FFRNT) against Omicron BA.1 and BA.4/BA.5 lineages [6–8]. For the ancestral strain, SARS-CoV-2 neutralizing geometric mean titers (GMTs) were measured in serum obtained before dose 1, 1 month after dose 2, and before and 1 month after dose 3 from the 2- or 3-dose immunogenicity set (defined in Supplementary Figure 1). For the FFRNT assessments against Omicron BA.1 and BA.4/BA.5 lineages, SARS-CoV-2 neutralizing GMTs were measured 1 month after dose 2 and 1 month after dose 3 in the Omicron neutralization subset (defined in Supplementary Figure 1). Of note, not all participants had blood samples at each of these time points. The ratio of GMTs (ie, geometric mean ratio [GMR]) and differences in percentages of participants with seroresponse (defined in the Appendix) 1 month after dose 3 to 1 month after dose 2, and geometric mean fold rises (GMFRs) in titers from before to after dose 3 were calculated. Further details regarding immunogenicity assessments and an overview of statistical analyses are summarized in the Appendix.

## RESULTS

All 401 enrolled participants received the third (booster) BNT162b2 dose at least 5 months after dose 2 (most commonly between 8 and 9 months after dose 2 [86.8%]) and comprise the safety population (see Supplementary Table S1 for demographic characteristics of the participants). The first open-label dose 3 was administered on January 31, 2022, at the time that the Omicron BA.1 subvariant was dominant in the United States. There were no withdrawals from the study by the immunogenicity cutoff date (March 22, 2022); at that time, 311 participants had completed the 1 month after dose 3 vaccination visit and 90 participants had not. Median (range) of follow-up after dose 3 was 1.3 (1.0−1.8) months.

**Figure 1.**
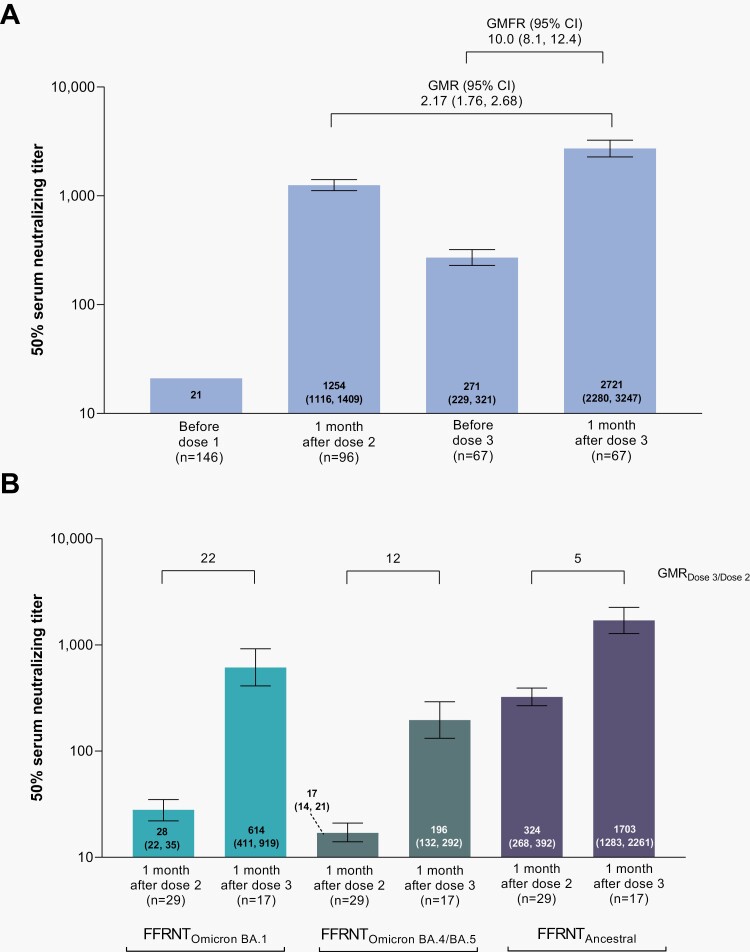
Serum SARS-CoV-2 neutralization titers 1 month after BNT162b2 doses 2 and 3 in participants without evidence of prior SARS-CoV-2 infection. In Panel A, 50% neutralizing titers were determined in a validated microneutralization assay against ancestral SARS-CoV-2 strain (USA-WA1/2020). Results are in the dose 2 and dose 3 evaluable immunogenicity populations (defined in Supplementary Figure 1). The *n* value for the before dose 1 timepoint is the total number of participants who were either dose 2 evaluable or dose 3 evaluable. Values within the bars are GMTs (95% CIs). The geometric mean ratio (GMR) shown is 1 month after dose 3 to 1 month after dose 2 and the geometric mean fold rise (GMFR) shown is from before dose 3 to 1 month after dose 3. Assay results below the lower limit of quantitation (LLOQ) of 41 were set to 0.5 × LLOQ. In Panel B, 50% serum neutralizing titers against ancestral SARS-CoV-2 and the Omicron BA.1 and BA.4/BA.5 sublineages are shown. Values within the bars are GMTs (95% CIs) and the GMR after dose 3 to after dose 2 are shown above the bars. Assay results below the LLOQ of 20 were set to 0.5 × LLOQ. Results are in the Omicron neutralization subset (defined in Supplementary Figure 1A) and based on the Fluorescent Focus Reduction Neutralization Test (FFRNT). Results in participants with and without prior SARS-CoV-2 infection are in Supplementary Figure 2.

A third BNT162b2 dose elicited ancestral SARS-CoV-2 neutralizing responses 1 month after dose 3 that were substantially increased versus those 1 month after dose 2, with a GMR of 2.17 (Figure 1A). The percentage of participants achieving seroresponse was 100% at 1 month after dose 2, decreasing to 77.6% before dose 3, and increasing to 98.5% 1 month after dose 3 (Supplementary Table 2).

In a subset of participants, Omicron sublineage BA.1 neutralizing GMTs were 28 and 614 at 1 month after dose 2 and 1 month after dose 3, respectively, corresponding to a 22-fold titer increase after the third dose (Figure 1B). Similarly, Omicron sublineage BA.4/BA.5 neutralizing GMTs were 17 and 196 at these respective time points, corresponding to a 12-fold titer increase after the third dose. Ancestral neutralizing GMTs 1 month after dose 2 and 1 month after dose 3 were 324 and 1703, respectively, corresponding to a 5-fold titer increase after the third dose.

**Figure 2.**
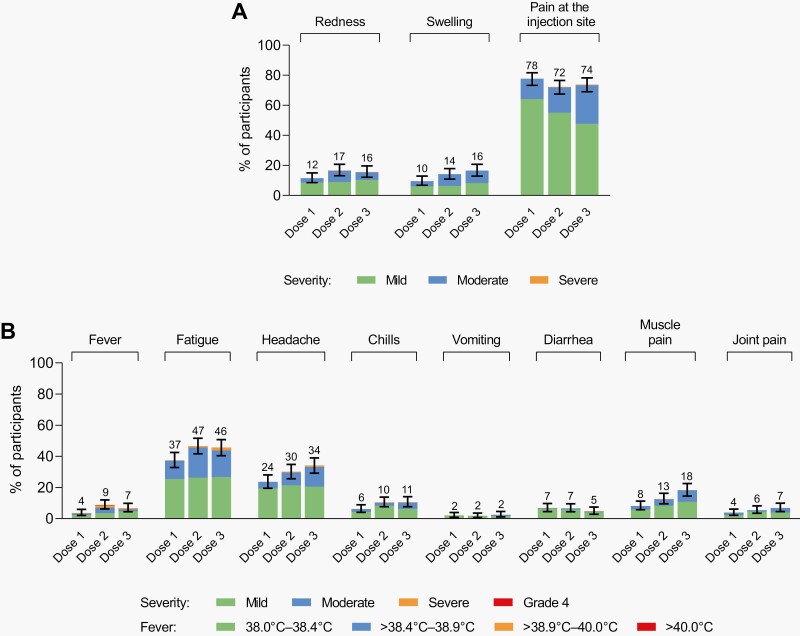
Local reactions and systemic events. Panel A shows local reactions and Panel B shows systemic events in BNT162b2 recipients with electronic-diary data after dose 1 (*n* = 398), dose 2 (*n* = 399), and dose 3 (*n* = 371). Grading of reactogenicity events is outlined in the Appendix. Numbers above the bars are the percentage of participants with that local reaction or systemic event with any severity within 7 days after each dose. One participant experienced fever of >40.0 °C after dose 2 (body temperature of 39.7 °C on day 2 and 40.3 °C on day 3). This participant also had a fever of 39.0 °C occurring 1 day after dose 3, which resolved within 3 days with the use of concomitant medication. Technical issues that impacted activation of the electronic diary after dose 3 resulted in 36 participants not recording events on day 1 after dose 3. For 11 of these participants, events were recorded by day 2. For the remaining 25 participants, study site staff contacted participants/parents/legal guardians regarding the occurrence of reactogenicity events; only 1 participant had fatigue and injection site pain.

The third BNT162b2 dose was well tolerated, with a safety profile consistent with previous doses in this age group. No new safety signals were identified. Most reactogenicity events were mild to moderate in severity and transient; no grade 4 events or fevers >40 °C were reported (Figure 2). Similar to doses 1 and 2, injection-site pain was the most common local reaction, and fatigue and headache were the most common systemic events.

Up to 1 month after dose 3, AEs were reported by 9% of participants and no AEs led to withdrawal (Supplementary Table 3). No serious AEs, deaths, or myocarditis/pericarditis or anaphylaxis cases were reported. Through the safety data cutoff (July 30, 2022), lymphadenopathy was reported in 15 participants (3.7%), including lymph node pain in 1 participant. Most cases of lymphadenopathy were mild and resolved within 1 week; 14 of 15 cases were considered vaccine-related.

## DISCUSSION

A third (booster) BNT162b2 10-µg dose administered approximately 6 months after the second dose to 5- to 11-year olds increases neutralizing titers against SARS-CoV-2, including against Omicron BA.1 and BA.4/BA.5 strains, and is expected to confer protection against COVID-19 including illness caused by Omicron variants. This is in the context of previously observed immunogenicity and efficacy results across adolescent and adult clinical trial populations and from real-world data, which have collectively shown that a third (booster) dose of BNT162b2 substantially increases the magnitude and breadth of neutralization and provides protection against symptomatic SARS-CoV-2 infection, including those caused by Omicron variants [2–5]. Consistent with recent data showing decreased antibody neutralization to Omicron BA.4/BA.5 compared to other Omicron subvariants after a third or fourth COVID-19 vaccine dose administered to adults [9, 10], the magnitude of neutralization after the third (booster) BNT162b2 dose in our study was lower for Omicron BA.4/BA.5 sublineages than for ancestral and Omicron BA.1 strains.

A third (booster) BNT162b2 10-µg dose administered to 5- to 11-year olds had an acceptable safety and tolerability profile, and no new safety concerns were identified. Reactogenicity after the third (booster) dose was mostly mild to moderate in severity, short-lived, and generally comparable to that observed after the two-dose series [1]. Lymphadenopathy frequency (3.7%) after dose 3 in 5- to 11-year olds was higher than previously observed after dose 2 of 10-µg BNT162b2 in this age group (0.9%), and similar to that observed after dose 3 of 30-µg BNT162b2 in ≥16-year olds (2.7%) [1, 5]. This pattern of increased lymphadenopathy after dose 3 is consistent in adults and children and likely attributable to the post-dose 3 boosted immune response. The safety profile of a third (booster) BNT162b2 dose in this age group is also supported by an assessment by the US Centers for Disease Control and Prevention, which included AEs reported to the Vaccine Adverse Event Reporting System and a voluntary safety surveillance system [11]. During the assessment period, more than 650,000 children received a third (booster) BNT162b2 dose. Reactogenicity events were similar in frequency after doses 2 and 3, serious AEs were rare, and no myocarditis or deaths were reported.

Limitations of this analysis include the short follow-up, lack of efficacy assessment, and data in children who had a longer interval between the primary series and dose 3 were not available. Long-term follow-up from this and other studies is ongoing.

These data contribute to evidence supporting benefits of a third (booster) BNT162b2 dose, which received emergency use authorization for 5- to 11-year olds in May 2022 [11]. More recently, in October 2022, a bivalent booster (composed of original BNT162b2 plus Omicron-BA.4/BA.5-adapted BNT162b2) was authorized in 5- to 11-year olds [12]. The authorization was based in part on the safety and immunogenicity data described here; this highlights the importance of clinical trial data alongside the need for agility in the face of the ongoing pandemic. Emerging data from clinical trials and real-world assessments will further clarify the effectiveness of two monovalent BNT162b2 doses followed by a bivalent booster, durability of vaccine-induced immune responses, and the need for additional dose(s) and additional variant-specific vaccine formulations in school-aged children.

## SUPPLEMENTARY DATA

Supplementary materials are available at the Journal of The Pediatric Infectious Diseases Society online (http://jpids.oxfordjournals.org).

piad015_suppl_Supplementary_Appendix

## Notes

***Potential conflicts of interest***. Eric A. F. Simões reports grants from Pfizer Inc and Regeneron Pharmaceuticals Inc. Nicola P. Klein reports grants from GlaxoSmithKline, Merck, Pfizer, Protein Science, and Sanofi Pasteur Inc. Benita Ukkonen and Piotr Korbal have no disclosures. Jing Zou, Xuping Xie, and Pei-Yong Shi report grants from Pfizer Inc. Ӧzlem Türeci and Uğur Şahin are employees of BioNTech Inc and may hold stock or stock options. Charu Sabharwal, Alejandra Gurtman, Nicholas Kitchin, Uzma N. Sarwar, Xia Xu, Stephen Lockhart, Luke Cunliffe, Claire Lu, Hua Ma, Kena A. Swanson, Kenneth Koury, David Cooper, Kathrin U. Jansen, and William C. Gruber are current or former employees of Pfizer and may hold stock or stock options.

***Data sharing statement***. Upon request, and subject to review, Pfizer will provide the data that support the findings of this study. Subject to certain criteria, conditions and exceptions, Pfizer may also provide access to the related individual de-identified participant data. See https://www.pfizer.com/science/clinical-trials/trial-data-and-results for more information.

***Financial support*.** This work was sponsored by BioNTech, funded by Pfizer.
