# Supplementary material for: Immunogenicity and Safety of a Third COVID-19 BNT162b2 mRNA Vaccine Dose in 5- to 11-Year Olds
Source: J Pediatric Infect Dis Soc. 2023 Mar 16;12(4):234–8. doi: 10.1093/jpids/piad015 (PMC10146923; doi:10.1093/jpids/piad015)

APPENDIX

List of Investigators 2
Eligibility Criteria 5
Ethical Conduct of the Study 5
Study Responsibilities 5
Procedures 5
Grading of Local Reactions and Systemic Events 6
Determination of Prior Infection Status 6
Definition of Seroresponse 6
Immunogenicity Analyses 6
Statistical Analysis 7
Table S1. Demographic characteristics* 8
Table S2. Seroresponse in participants without evidence of prior infection 9
Table S3. Participants reporting adverse events 10
Figure S1. Study populations 11
Figure S2. Serum SARS-CoV-2 neutralization titers 1 month after BNT162b2 doses 2 and 3 in participants regardless of prior SARS-CoV-2 infection 12

List of Investigators

Name	Institute	Location
Acevedo, Armando	Acevedo Clinical Research Associates	Miami, FL, USA
Amisola, Rogelio	PriMed Clinical Research	Dayton, OH, USA
Anderson, Evan	Emory Children's Center Illness POD	Atlanta, GA, USA
	Emory University School of Medicine	Atlanta, GA, USA
Armiñana, Angels Ulied	EAP Centelles	Barcelona, Spain
Arora, Samir	Aventiv Research Inc	Columbus, OH, USA
Baker, Jeffrey	Clinical Research Prime	Idaho Falls, ID, USA
Barnett, Elizabeth	Boston Medical Center	Boston, MA, USA
Boppana, Suresh	University of Alabama at Birmingham - School of Medicine	Birmingham, AL, USA
Butuk, David	Solaris Clinical Research	Meridian, ID, USA
Caserta, Mary	University of Rochester Medical Center	Rochester, NY, USA
Casey, Janet	Rochester Clinical Research, Inc.	Rochester, NY, USA
Caviness, Alison (previously Benun, Jacques)	ARC Clinical Research at Wilson Parke	Austin, TX, USA
Christensen, Shane	J. Lewis Research, Inc / Foothill Family Clinic South	Salt Lake City, UT, USA
Cots, Manuel Baca	Grupo Pediatrico Uncibay	Malaga, Spain
Czajka, Hanna	Centrum Badań Klinicznych Jagiellońskie	Krakow, Poland
Day, James (previously Pahud, Barbara)	Children's Mercy Hospital	Kansas City, MO, USA
de la Cueva, Ignacio Salamanca	Instituto Hispalense de Pediatria	Sevilla, Spain
De Valle, Oscar	West Houston Clinical Research Service	Houston, TX, USA
Dever, Michael	Clinical Neuroscience Solutions	Orlando, FL, USA
Domachowske, Joseph	SUNY Upstate Medical University	Syracuse, NY, USA
Eder, Frank	Meridian Clinical Research, LLC	Binghamton, NY, USA
Englund, Janet	Seattle Children`s Hospital	Seattle, WA, USA
Fergie, Jaime	Driscoll Children's Hospital	Corpus Christi, TX, USA
Finn, Daniel	Kentucky Pediatric / Adult Research	Bardstown, KY, USA
Garza, Homero	Van Tran Family Practice	Houston, TX, USA
	Ventavia Research Group, LLC	Houston, TX, USA
Guasch, Claudia Fortuny	Hospital Sant Joan de Deu	Barcelona, Spain
Hand, Jonathan (previously Garcia-Diaz, Julia)	Ochsner Clinic Foundation	New Orleans, LA, USA
Hartman, Aaron	Virginia Research Center	Midlothian, VA, USA
Heller, Robert	Bayview Research Group, LLC	Valley Village, CA, USA
Jennings, Timothy	Clinical Research Professionals	Chesterfield, MO, USA
Kantele, Anu	MeVac, Meilahti Vaccine Research Center	Helsinki, Finland

Klein, Nicola	Kaiser Permanente Oakland	Oakland, CA, USA
	Kaiser Permanente Sacramento	Sacramento, CA, USA
	Kaiser Permanente Santa Clara	Santa Clara, CA, USA
Klein, Terry	Alliance for Multispecialty Research, LLC	Wichita, KS, USA
Kokko, Satu	Tampere University/Oulu Vaccine Research Clinic	Oulu, Finland
Kopinska, Elzbieta	Nasz Lekarz Osrodek Badan Klinicznych	Torun, Poland
Korbal, Piotr	IN VIVO Spolka z ograniczona	Bydgoszcz, Poland
Koski, Susanna	Tampere University/Helsinki South Vaccine Research Clinic	Helsinki, Finland
Koski, Susanna (previously Sirnela-Rif, Elina)	Tampere University/Helsinki East Vaccine Research Clinic	Helsinki, Finland
Kuchar, Ernest	Provita 001	Warszawa, Poland
Kulig, Karolina	Osrodek Badan Klinicznych Appletreeclinics	Lodz, Poland
Laajalahti, Outi	Tampere University/Seinäjoki Vaccine Research Clinic	Seinäjoki, Finland
Leonard, Daniel	Meridian Clinical Research, LLC	Hastings, NB, USA
Li, Simon	Rutgers University	New Brunswick, NJ, USA
Lopez, Jose Garcia-Sicilia	Hospital Universitario HM Monteprincipe	Madrid, Spain
Majda-Stanislawska, Ewa	GRAVITA Diagnostyka i Leczenie nieplodnosci	Lodz, Poland
Maldonado, Yvonne	Clinical & Translational Research Unit (CTRU) & Spectrum BioBank, Stanford University	Palo Alto, CA, USA
Marshall, Gary	Novak Center for Children's Health	Louisville, KY, USA
Martinez, Silvina Laura Natalini	Hospital HM Puerta del Sur	Mostoles, Spain
Montaña, Josep-Lluis Arimany	Hospital Universitari General de Catalunya	Barcelona, Spain
Muñoz, Flor	Texas Children's Hospital - Clinical Research Center	Houston, TX, USA
Nachman, Sharon	Advanced Specialty Care	Commack, NY, USA
	Clinical Research Center	East Setauket, NY, USA
	Stony Brook University	Stony Brook, NY, USA
Ogbuagu, Onyema	Yale Center for Clinical Investigation	New Haven, CT, USA
Ohnmacht, Richard	Velocity Clinical Research-Providence	East Greenwich, RI, USA
Paavola, Pauliina	Tampere University/Kokkola Vaccine Research Clinic	Kokkola, Finland
Pajek, Barbara	Niepubliczny Zaklad Lecznictwa	Siemianowice Slaskie, Poland
Patel, Nehali	St. Jude Children's Research Hospital	Memphis, TN, USA
Paulsen, Grant	Cincinnati Children's Hospital Medical Center	Cincinnati, OH, USA
Peltonen, Lauri	Tampere University/Jarvenpaa Vaccine Research Clinic	Jarvenpaa, Finland
Peterson, James	J. Lewis Research, Inc. / Foothill Family Clinic South	Salt Lake City, UT, USA
Rey, Ma Mildred	Center for Clinical Trials	Paramount, CA, USA
	Center for Clinical Trials, LLC	Paramount, CA, USA
Riesenberg, Robert	Atlanta Center for Medical Research	Atlanta, GA, USA

Rojo, Pablo	Hospital Universitario 12 de Octubre	Madrid, Spain
Senders, Shelly	Senders Pediatrics	South Euclid, OH, USA
Seppa, Ilkka	Tampere University/Turku Vaccine Research Clinic	Turku, Finland
Seppa, Ilkka (previously Karhusaari, Hanna)	Tampere University/Pori Vaccine Research Clinic	Pori, Finland
Seppa, Ilkka (previously Sipila, Marjaana)	Tampere University/Tampere Vaccine Research Clinic	Tampere, Finland
Sharp, Stephan	Clinical Research Associates Inc	Nashville, TN, USA
Sher, Lawrence	Peninsula Research Associates	Rolling Hills Estates, CA, USA
Simões, Eric	Children's Hospital Colorado	Aurora, CO, USA
Simonsen, Kari	Children's Hospital & Medical Center	Omaha, NB, USA
	Children's Physician's Clinic, Spring Valley	Omaha, NB, USA
Slade, Lance	Meridian Clinical Research, LLC	Macon, GA, USA
Slechta, Stacy	Alliance for Multispecialty Research, LLC	Newton, KS, USA
Stryczynska-Kazubska, Joanna	Rodzinne Centrum Medyczne LUBMED	Lubon, Poland
Talaat, Kawsar	Johns Hopkins Bayview Medical Center	Baltimore, MD, USA
Torres, Federico Martinon	CHUS - Hospital Clinico Universitario	Santiago de Compostela, Spain
Ukkonen, Benita	Tampere University/Espoo Vaccine Research Clinic	Espoo, Finland
Valdivieso, Mariano Miranda	Hospital de Antequera	Antequera, Spain
Vanchiere, John	Louisiana State University Health Sciences	Shreveport, LA, USA
Walter, Emmanuel	Duke Vaccine and Trials Unit	Durham, NC, USA
Wiedermann, Bernhard	Children`s National Medical Center	Washington, DC, USA
Wisman, Paul	Pediatric Associates of Charlottesville, PLC	Charlottesville, VA, USA
Zomcik, Anne	AHN Erie Health + Wellness Pavilion: West	Erie, PA, USA

Eligibility Criteria

Healthy 5–11-year-old participants with pre-existing stable disease could be included, defined as disease not requiring a significant change in therapy or hospitalization for worsening disease during the 6 weeks before enrollment. Participants with chronic stable HIV or hepatitis B or C infection could be included. Receipt of COVID-19 preventative treatments, previous or current diagnosis of multisystem inflammatory syndrome in children (MIS-C), history of severe adverse reaction to a vaccine or any component of the study intervention, and immunodeficiency, autoimmune disease, or conditions associated with prolonged bleeding were exclusion criteria. Full eligibility criteria for the study, including the study protocol, are reported elsewhere.[1]

Ethical Conduct of the Study

Conduct of the study was in accordance with the study protocol and with consensus ethical principles derived from international guidelines, including the Declaration of Helsinki, International Conference on Harmonisation Guidelines for Good Clinical Practice, CIOMS International Ethical Guidelines, and applicable laws and regulations, including applicable privacy laws. The study protocol and any amendments, informed consent documents, and other relevant documents were approved by institutional review boards or ethics committees before study initiation. Before any study activity, written informed consent from the parent/guardian (and verbal or written participant assent if applicable) was obtained. The parent/legal guardian as applicable were also reconsented to the most current version of the informed consent document/assent during their participation in the study.

Study Responsibilities

Pfizer was responsible for study design and conduct; data collection, analysis, and interpretation; and writing of this manuscript. Both Pfizer and BioNTech manufactured the vaccine. BioNTech was the regulatory sponsor of the studies and contributed to data interpretation and writing of the manuscript. All data were available to the authors, who vouch for its accuracy and completeness and to adherence to the study protocols.

Procedures

In this study, existing 5–11-year-old participants from the phase 2/3 study who received two 10 µg BNT162b2 doses in the phase 2/3 study received an open-label third (booster) dose of 10 µg BNT162b2 ≥6 months after the second dose. The dose level of the second and third BNT162b2 doses was based on the participant’s age at the time of vaccination. Therefore, participants who were 12-years-old at the time of receiving the third dose were not included in this analysis as they received the recommended age-appropriate 30-ug dose level.

[1] Walter EB, Talaat KR, Sabharwal C, et al. N Engl J Med. 2022;386:35-46. Available at: https://www.nejm.org/doi/full/10.1056/nejmoa2116298.

Grading of Local Reactions and Systemic Events

Pain at the injection site was graded as mild (does not interfere with activity), moderate (interferes with activity), severe (prevents daily activity), or grade 4 (led to an emergency department visit or hospitalization). Redness and swelling were graded as mild (0.5–2.0 cm in diameter), moderate (>2.0–7.0 cm in diameter), severe (>7.0 cm in diameter), or grade 4 (necrosis or exfoliative dermatitis for redness and necrosis for swelling). Fatigue/tiredness, headache, chills, new or worsened muscle pain, and new or worsened joint pain were graded as mild (does not interfere with activity), moderate (some interference with activity), or severe (prevents daily routine activity). Vomiting was graded as mild (1–2 times in 24 hours), moderate (>2 times in 24 hours), or severe (requires intravenous hydration) and diarrhea as mild (2–3 loose stools in 24 hours), moderate (4–5 loose stools in 24 hours) or severe (≥6 loose stools in 24 hours). Grade 4 for all systemic events indicated an emergency department visit or hospitalization. Fever (ie, ≥38.0ºC) was graded as ≥38.0ºC–38.4ºC, >38.4ºC–38.9ºC, >38.9ºC–40.0ºC, and >40.0ºC.

Determination of Prior Infection Status

Prior infection status was determined by serologic testing (SARS-CoV-2 N-binding assay) for immunoglobulin to the SARS-CoV-2 N-antigen at baseline (dose 1 visit), 1 month after dose 2, dose 3, and 1 month after dose 3, and with a SARS-CoV-2 nucleic acid amplification test (NAAT) on anterior nares swabs at dose 1, dose 2, and dose 3, unscheduled visits, and medical history of COVID-19.

Definition of Seroresponse

Seroresponse was defined as a ≥4-fold rise from before dose 1, or ≥4 × lower limit of quantitation (LLOQ) if the baseline measurement was <LLOQ.

Immunogenicity Analyses

Immunogenicity analyses were conducted with the validated neutralization assay[2] for an immunogenicity set of participants based on the evaluable immunogenicity populations. The immunogenicity set was comprised of the 3-dose set, which included the first 130 participants who received dose 3 and completed the 1 month after dose 3 visit before March 15, 2022 (defined in **Figure S1A**). Within this set, 30 participants also had blood sample collection at 1 month after dose 2. The 2-dose set included 67 additional participants randomly selected from the previously analyzed dose 2 evaluable immunogenicity population who were without evidence of prior infection ≤1 month after dose 2. These additional participants were included to ensure sufficient data for analyses were

[2] Walsh EE, Frenck RW Jr, Falsey AR, et al. N Engl J Med 2020;383:2439-2450.

obtained 1 month after dose 2. The dose 2 and dose 3 evaluable immunogenicity populations are defined in **Figure S1A** and data from this set are included in **Figure 1A**.

Immunogenicity analyses were also conducted on the Omicron neutralization subset (defined in **Figure S1A**). Analyses were conducted for participants without evidence of prior SARS-CoV-2 infection (results from this subset are shown in **Figure 1A**) and in participants with and without prior SARS-CoV-2 infection (results from this subset are shown in **Figure S2B**). A non-validated Fluorescent Focus Reduction Neutralization Test (FFRNT)[3,4] was used to determine SARS-CoV-2 serum neutralizing titers 1 month after dose 2 and 1 month after dose 3. FFRNT GMTs were obtained from the 50% FFRNT titers determined against the designated wild-type reference strain (recombinant USA-WA1/2020) and against the Omicron BA.1 sublineage, which is a recombinant virus with the Omicron BA.1 spike sequence on the genetic background of USA-WA1/2020. All samples were tested at the same time to ensure comparability of results.

Statistical Analysis

Safety endpoints are presented descriptively for the safety population (ie, all participants who received a third dose of BNT162b2 by February 22, 2022) as counts, percentages, and associated Clopper–Pearson 2-sided 95% CIs. Adverse events were coded using the *Medical Dictionary for Regulatory Activities* v24.1 term for each group.

GMTs were obtained by exponentiating the mean of logarithmically transformed neutralizing titer values. Associated 2-sided 95% CIs were obtained based on the Student *t* distribution and then the confidence limits were exponentiated to express results on the original scale. Geometric mean ratios were obtained by exponentiating the mean of the differences ([dose 3 group at the 1 month after dose 3 time point] – [dose 2 group at the 1 month after dose 2 time point]) of logarithmically transformed neutralizing titer values. Associated 2-sided 95% CIs were based on the Student *t* distribution and then exponentiating results. GMFRs were calculated for participants by exponentiating the logarithm of fold rises. Associated 2-sided 95% CIs were obtained for the mean difference using the Student *t* distribution and the confidence limits were exponentiated. The exact 2-sided 95% CI for the percentage of participants with a seroresponse was computed using the F distribution (Clopper-Pearson) and the 2-sided 95% CI for the difference in seroresponse was determined using the Miettinen and Nurminen method.

[3] Zou J, Xia H, Xie X, et al. Nat Commun 2022;13:852.
[4] Kurhade C, Zou J, Xia H, et al. Nat Commun 2022;13:3602.

Table S1. Demographic characteristics*

Characteristic	BNT162b2 (N=401)
Sex, n (%)	
Male	210 (52.4)
Female	191 (47.6)
Race, n (%)†	
White	281 (70.1)
Black or African American	29 (7.2)
Asian	31 (7.7)
Multiracial	46 (11.5)
American Indian or Alaska Native	8 (2.0)
Other/not reported	6 (1.5)
Ethnicity, n (%)†	
Hispanic/Latinx	92 (22.9)
Not reported	3 (0.7)
Country, n (%)	
USA	401 (100.0)
Age at first dose vaccination, y	
Mean (standard deviation)	7.9 (1.75)
Median (range)	8.0 (5–11)
Baseline SARS-CoV-2 status (at the time of dose 1), n (%)‡	
Positive	22 (5.5)
Negative	379 (94.5)
Comorbidities, n (%)§¶	
Yes	119 (29.7)
No	282 (70.3)
Obese, n (%)#	39 (9.7)

* Results are for the safety population (defined in **Figure S1**). Percentages may not total 100 because of rounding.

† Race and ethnic group were reported by the parent/guardian.

‡ Baseline SARS-CoV-2 positive required a positive N-binding antibody or positive nucleic acid amplification test (NAAT) result at dose 1, or a medical history of COVID-19.

§ Participants with ≥1 comorbidity that increases the risk of severe COVID-19 (defined as participants with ≥1 prespecified comorbidity as outlined by COVID-NET[5] and/or obesity as defined in #).

¶ In the current study, the only medical history associated with increased risk of severe COVID-19 as outlined by COVID-NET[5] (ie, asthma, congenital heart disease, diabetes mellitus, feeding tube dependent, immunocompromised condition, neurologic disorder, obesity, premature infant, sickle cell disease) was asthma (n=31; 7.7%), obesity (n=2; 0.5%), premature infant (n=2; 0.5%), and sickle cell trait (n=1; 0.2%).

# Body mass index at or above the 95th percentile according to the US Centers for Disease Control and Prevention growth chart (https://www.cdc.gov/growthcharts/html_charts/bmiagerev.htm).

[5] Kim L, Whitaker M, O'Halloran A, et al. MMWR Morb Mortal Wkly Rep. 2020;69(32):1081-1088.

Table S2. Seroresponse in participants without evidence of prior infection

Assay	Time point	n/N	Seroresponse*, % (95% CI)	Difference† (95% CI)
SARS-CoV-2 neutralization assay	1 month after dose 2	96/96	100.0 (96.2, 100.0)	
	Before dose 3	52/67	77.6 (65.8, 86.9)	–1.5 (–8.0, 2.4)
	1 month after dose 3	66/67	98.5 (92.0, 100.0)	

Results are for the evaluable immunogenicity populations (defined in **Figure S1**).
Participants included in this analysis had no serological or virological evidence of past SARS-CoV-2 infection for up to 1 month after dose 2 (for the 1 month after dose 2 time point) or 1 month after dose 3 (for the before dose 3 and 1 month after dose 3 time points) study blood sample collection. Having no evidence of past SARS-CoV-2 infection up to 1 month after dose 2 was defined as having a negative N-binding antibody (serum) result at the dose 1 and 1 month after dose 2 study visits; a negative NAAT (nasal swab) result at the dose 1 and dose 2 study visits and any unscheduled visit before the 1 month after dose 2 blood sample collection; and no medical history of COVID-19. Having no evidence of past SARS-CoV-2 infection ≤1 month after dose 3 was defined as having a negative N-binding antibody (serum) result at the dose 1, 1 month after dose 2 (if available), dose 3, and 1 month after dose 3 study visits; a negative NAAT (nasal swab) result at the dose 1, dose 2, and dose 3 study visits and any unscheduled visit before the 1 month after dose 3 blood sample collection; and no medical history of COVID-19.
The data cutoff date was March 22, 2022.
* Seroresponse was defined as achieving a ≥4-fold rise from baseline (before dose 1). If the baseline measurement was below the lower limit of quantification (LLOQ), a post-vaccination assay result ≥4 × LLOQ was considered a seroresponse.
† The difference in proportions, expressed as a percentage, was calculated as ([1 month after dose 3 group at the 1 month after dose 3 time point] – [1 month after dose 2 group at the 1 month after dose 2 time point]). The 1 month after dose 3 group includes participants in the 3-dose immunogenicity set who had assay results at 1 month after dose 3 and the 1 month after dose 2 group includes participants in the 2-dose or 3-dose immunogenicity set who had assay results 1 month after dose 2.

Table S3. Participants reporting adverse events

Adverse event	BNT162b2 (N*=401) n† (%)
Any event (from dose 3 to 1 month after dose 3)	36 (9.0)
Related§	19 (4.7)
Severe¶	1 (0.2)
Life-threatening	0
Any serious adverse event (from dose 3 to safety data cutoff)	2 (0.5)
Related§	0
Any adverse event leading to discontinuation (from dose 3 to safety data cutoff)	0
Death (from dose 3 to data cutoff)	0
Adverse events of clinical interest (from dose 3 to safety data cutoff)#	
Lymphadenopathy**	15 (3.7)
Rash††	1 (0.2)

Results are for the safety population (defined in **Figure S1**).
The safety data cutoff date was July 30, 2022.
* Number of participants in the specified group. This value is the denominator for the percentage calculations.
† Number of participants reporting ≥1 occurrence of the specified event category. For 'any event', n is the number of participants reporting ≥1 occurrence of any event.
§ Assessed by the investigator as related to the investigational product, the majority of which were reactogenicity events.
¶ Grade 3 fever of 39.0ºC in a 5-year-old participant occurred at Day 1 after dose 3, resolved within 3 days with the use of concomitant medication, and was considered to be vaccine-related.
# The following Standardized MedDRA Queries (SMQs) were applied to search the safety data: angioedema, arthritis, convulsions, demyelination, hypersensitivity, peripheral neuropathy, and vasculitis. There were no preferred terms associated with these SMQs, except for 1 case of rash. Additionally, there were no adverse events of clinical interest reported for anaphylaxis, myocarditis, pericarditis, Bell's palsy (or facial paralysis/paresis), or appendicitis. Other notable adverse events in which no cases were reported in this population as of the data cutoff included (but were not limited to): arthritis, thrombocytopenic events, thromboembolic or intravascular coagulation events, autoimmune or demyelination events, meningitis, encephalitis, neuritis, peripheral neuropathy, vasculitis, Kawasaki disease, MIS-C, or acute respiratory distress syndrome. The only AEs of clinical interest in this safety dataset was lymphadenopathy.
**Lymphadenopathy included lymph node pain and was considered vaccine-related by the investigator in 14 participants (3.5%); most had an onset within 2 days of dose 3 and resolved approximately 1 week after onset.
†† 1 case of rash, which was not considered related to vaccination by the investigator, had an onset of 11 days after dose 3, and resolved within 4 days.

Figure S1. Study populations

Population	Definition	N	Notes
Safety	All participants who received a third 10-µg BNT162b2 dose by February 22, 2022	401	
3-dose immunogenicity set	The first 130 participants who received dose 3 and completed the 1 month after dose 3 visit before March 15, 2022	130	Among these, 30 participants also had blood sample collection at the 1 month after dose 2 visit (the population included in **Table S2**)
2-dose immunogenicity set	Included up to 70 additional participants randomly selected from the previously analyzed dose 2 evaluable immunogenicity population who were without evidence of prior infection up to 1 month after dose 2	67	(The population included in **Table S2**)
Dose 2 evaluable immunogenicity	Participants who received all 2 vaccine doses at the same dose level as randomized within the protocol specified window for each dose, having ≥1 valid and determinate immunogenicity result within 28–42 days after dose 2, and not having any important protocol deviations affecting evaluability	96	Without evidence of prior SARS-CoV-2 infection up to 1 month after dose 2 (the population included in **Figure 1A** for the 1 month after dose 2 timepoint)
Dose 3 evaluable immunogenicity	Participants who received all 3 vaccine doses at the same dose level as randomized within the protocol specified window for each dose, having ≥1 valid and determinate immunogenicity result within 28–42 days after dose 3, and not having any important protocol deviations affecting evaluability	67	Without evidence of prior SARS-CoV-2 infection up to 1 month after dose 3 (the population included in **Figure 1A** for the before dose 3 and the 1 month after dose 3 timepoints)
Dose 2 or dose 3 evaluable immunogenicity	Participants who were in the dose 2 evaluable immunogenicity or dose 3 evaluable immunogenicity population	146	Without evidence of prior SARS-CoV-2 infection up to 1 month after dose 2 (the population included in **Figure 1A** for the before dose 1 timepoint)
Dose 2 all-available immunogenicity	All randomized participants who received 2 vaccine doses and with ≥1 valid and determinate immunogenicity result at 1 month after dose 2	97	Regardless of prior SARS-CoV-2 infection (the population included in **Figure S2A** for the 1 month after dose 2 timepoint)
Dose 3 all-available immunogenicity	All randomized participants who received 3 vaccine doses and with ≥1 valid and determinate immunogenicity result at 1 month after dose 3	118	Regardless of prior SARS-CoV-2 infection (the population included in **Figure S2A** for the before dose 3 and 1 month after dose 3 timepoints)
Dose 2 or dose 3 all-available immunogenicity	All randomized participants who received the third BNT162b2 dose and with ≥1 valid and determinate immunogenicity result after vaccination	185	Regardless of prior SARS-CoV-2 infection up to 1 month after dose 3 (the population included in **Figure S2A**)
Omicron neutralization subset	Composed of up to 30 participants in the 3-dose set who had blood sample collection both at 1 month after dose 2 and 1 month after dose 3	30	Regardless of prior SARS-CoV-2 infection (the population included in **Figure S2B**)
		29	Without evidence of prior SARS-CoV-2 infection (the population included in **Figure 1B** for the 1 month after dose 2 timepoint)
		17	Without evidence of prior SARS-CoV-2 infection up to 1 month after dose 3 (the population included in **Figure 1B** for the 1 month after dose 3 timepoint)

Figure S2. Serum SARS-CoV-2 neutralization titers 1 month after BNT162b2 doses 2 and 3 in participants regardless of prior SARS-CoV-2 infection

In Panel A, 50% neutralizing titers were determined in a validated microneutralization assay against ancestral SARS-CoV-2 strain (USA-WA1/2020). Values within the bars are GMTs (95% CIs). The geometric mean ratio (GMR) shown is 1 month after dose 3 to 1 month after dose 2 and the geometric mean fold rise (GMFR) shown is from before dose 3 to 1 month after dose 3. Assay results below the lower limit of quantitation (LLOQ) of 41 were set to 0.5 × LLOQ. In Panel B, 50% serum neutralizing titers against ancestral SARS-CoV-2 and the Omicron BA.1 and BA.4/BA.5 sublineages are shown. Values within the bars are GMTs (95% CIs) and the GMR of after dose 3 to after dose 2 are shown above the bars. Assay results below the LLOQ of 20 were set to 0.5 × LLOQ. Results are based on the Fluorescent Focus Reduction Neutralization Test (FFRNT). Results in Panel A and Panel B are in the all-available immunogenicity population (defined in **Figure S1)**. n=number of participants with valid and determinate assay results for the specified assay at the given dose/sampling time point.

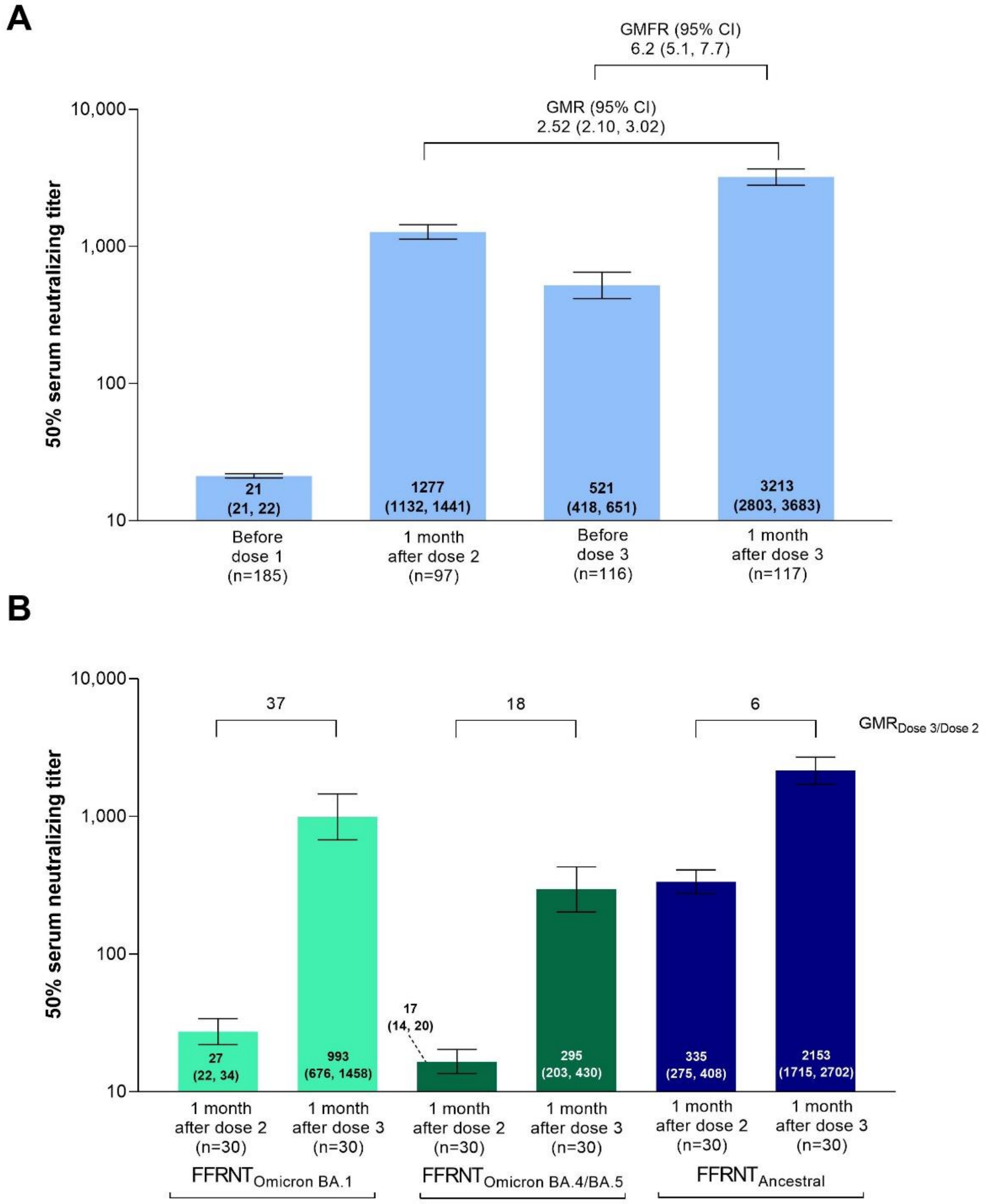

Supplement: piad015_suppl_Supplementary_Appendix [file piad015_suppl_supplementary_appendix.pdf]
